# Functional Components of Cognitive Impairment in Multiple Sclerosis: A Cross-Sectional Investigation

**DOI:** 10.3389/fneur.2017.00643

**Published:** 2017-11-28

**Authors:** Jordi A. Matias-Guiu, Ana Cortés-Martínez, María Valles-Salgado, Celia Oreja-Guevara, Vanesa Pytel, Paloma Montero, Teresa Moreno-Ramos, Jorge Matias-Guiu

**Affiliations:** ^1^Department of Neurology, Instituto de Investigación Sanitaria del Hospital Clínico San Carlos, Universidad Complutense de Madrid, Madrid, Spain

**Keywords:** multiple sclerosis, cognitive dysfunction, neuropsychological tests, fatigue, memory

## Abstract

**Background:**

Cognitive impairment is frequent and disabling in multiple sclerosis (MS). Changes in information processing speed constitute the most important cognitive deficit in MS. However, given the clinical and topographical variability of the disease, cognitive impairment may vary greatly and appear in other forms in addition to slower information processing speed. Our aim was to determine the frequency of cognitive impairment, the principal cognitive domains, and components involved in MS and to identify factors associated with presence of cognitive impairment in these patients in a large series of patients.

**Methods:**

Cross-sectional study of 311 patients with MS [236 with relapsing-remitting MS (RRMS), 52 with secondary progressive MS (SPMS), and 23 with primary progressive MS (PPMS)]. Patients’ cognitive function was assessed with a comprehensive neuropsychological assessment protocol. Patients displaying deficits in 2 or more cognitive domains were considered to have cognitive impairment associated with MS. We conducted a principal component analysis to detect different cognitive patterns by identifying clusters of tests highly correlated to one another.

**Results:**

Cognitive impairment was detected in 41.5% of the sample, and it was more frequent in patients with SPMS and PPMS (*P* = 0.002). Expanded Disability Status Scale scores and education were independent predictors of cognitive impairment. Principal component analysis identified seven clusters: attention and basic executive function (including information processing speed), planning and high-level executive function, verbal memory and language, executive and visuospatial performance time, fatigue-depression, visuospatial function, and basic attention and verbal/visual working memory. Mean scoring of components 2 (high-order executive functioning) and 3 (verbal memory-language) was higher in patients with RRMS than in those with PPMS (component 2) and SPMS (component 3).

**Conclusion:**

MS is linked to multiple cognitive profiles and disturbances in different domains. This suggests that cognitive alterations in MS are heterogeneous and affect other domains in addition to information processing speed.

## Introduction

Multiple sclerosis (MS) is an autoimmune inflammatory disease of the central nervous system that causes inflammatory lesions in the brain and spinal cord, leading to blood–brain barrier disruption, demyelination, and axonal damage. The frequency of cognitive deficits in MS varies among studies due to differences in diagnostic criteria, the characteristics of the control group, and the tests used for neuropsychological assessment (1). In any case, cognitive deficits are frequent in some of these patients (40–70%), and they have a major impact on MS: cognitive impairment has been correlated with performance of daily living activities, maintaining employment, quality of life, white matter lesion load, and atrophy detected by MRI (2, 3).

Different studies postulate that the pattern of cognitive impairment in MS is characterized by reduced information processing speed and suggest that this is the most relevant cognitive deficit in MS (4, 5). This has led researchers to measure cognitive impairment with tests assessing information processing speed, especially the Symbol Digit Modalities Test (SDMT) and the Paced Auditory Serial Addition Test (PASAT). These tests have been included in such test batteries as the Brief Repeatable Battery of Neuropsychological Tests (BRB-N), the Brief International Cognitive Assessment for Multiple Sclerosis (BICAMS), and the Minimal Assessment of Cognitive Function in MS (MACFIMS) (6–10). Rao’s BRB-N was first validated with good psychometric and diagnostic properties, although it did not have any test of visuospatial and executive functions. Subsequently, MACFIMS was developed, including a more comprehensive assessment of cognitive functions. Because time of administration of MACFIMS is about 90–120 min, a shorter version named BICAMS was proposed (Table 1 summarizes test and cognitive domains evaluated with each of those batteries). However, although these test batteries are certainly useful in clinical practice (11), assessing cognitive function cannot be based solely on the evaluation of a few cognitive domains or specific tasks. In this regard, impairment of other cognitive functions has been also emphasized. Cognitive domains displaying the most severe impairment in MS are information processing speed, attention-executive function, and memory (1, 12). Furthermore, other factors such as depression, fatigue, and physical disability must be adequately controlled due to the potential influence in neuropsychological testing. On the other hand, the literature shows heterogeneous results for the frequency of cognitive impairment in relapsing-remitting (RRMS), secondary progressive (SPMS), and primary progressive MS (PPMS) (13–15), and for the type of dysfunction associated with each clinical form.

**Table 1:** Main neuropsychological batteries currently used in multiple sclerosis (MS).

Cognitive domains	BRN-B	MACFIMS	BICAMS
Learning and memory	SRT, SPART	CVLT-II, BVMT-R	CVLT-II, BVMT
Complex attention (mainly information processing speed)	PASAT, SDMT	SDMT, PASAT	SDMT
Language (only verbal fluency)	COWAT	COWAT	–
Executive functioning	–	D-KEFS sorting test	–
Visuospatial and perceptual function	–	JLO	–
Social cognition	–	–	–

*BICAMS, Brief International Cognitive Assessment for MS; BRN-B, brief repeatable neuropsychological battery; BVMT-R, Brief Visuospatial Memory Test revised; COWAT, Controlled Oral Word Association Test; CVLT-II, California Verbal Learning Test second edition; D-KEFS, Delis–Kaplan Executive Function Scale; JLO, Judgment of Line Orientation; MACFIMS, minimal assessment of cognitive function in MS; PASAT, Paced Auditory Serial Addition Test; SDMT, Symbol Digit Modalities Test; SPART, Spatial Recall Test; SRT, Selective Reminding Test*.

In this regard, recently Planche et al. studied a group of 101 patients with MS classified into RRMS, SPMS, and PPMS. After controlling for several factors, SPMS were more impaired than RRMS in information processing speed, executive functions, verbal fluency, verbal episodic memory, and visuospatial construction; PPMS were more frequently impaired in verbal fluency than RRMS; and visuospatial construction was more frequently impaired in SPMS than PPMS (13). These findings raise the possibility of the existence of several cognitive profiles according to some clinical factors, such as the clinical form of MS.

Another interesting question is about the factors associated to cognitive impairment. Some studies have linked the duration of the illness, the degree of disability, or cognitive reserve, sometimes with heterogeneous results (16–18). The knowledge of factors associated to cognitive impairment is relevant in order to disentail the pathophysiology of cognitive impairment in MS, as well as to detect some groups of patients in which cognitive impairment should be more specifically investigated.

The aims of the study were three. First, to determine the extent of cognitive impairment in MS. Second, to identify the principal cognitive domains and components involved in cognitive impairment in MS. To this end, we performed a principal component analysis in a large series of patients classified by clinical form of MS (RRMS, SPMS, and PPMS) and who underwent a thorough cognitive assessment. This type of analysis has been used in other neurodegenerative diseases to detect different cognitive patterns by identifying clusters of test items correlating highly to one another (19), and in MS to assess the validity of MACFIMS (20, 21). And third, we aimed to find factors associated with the presence of cognitive impairment in these patients.

## Materials and Methods

### Study Design and Patient Sample

We conducted a cross-sectional study including 357 patients between June 2015 and February 2017. The study was approved by the Research Ethics Committee at our hospital (Study code 15/514-E). Patients were recruited from a MS Section of a tertiary hospital, which is a reference unit in the Region of Madrid, Spain. All participating patients agreed to participate in the study. We established the following inclusion criteria: (1) diagnosis of MS according to the revised McDonald criteria (22) and (2) ages between 18 and 80 years. Exclusion criteria were as follows: (1) patients who were deemed unable to undergo prolonged cognitive evaluation; (2) patients who had experienced a relapse within the previous 2 months or those taking corticosteroids; (3) presence of systemic, or developmental disorders potentially affecting cognition; (4) history of alcohol or drug abuse; (5) major depressive disorder at the time of inclusion; (6) neuropsychiatric disorders not attributable to MS; and (7) a history of other neurological diseases or trauma.

### Neuropsychological Assessment

Cognitive function was assessed with a battery including the following tests: forward and backward digit span, Corsi block-tapping test, parts A and B of the Trail Making Test (TMT), SDMT (written), Boston Naming Test (BNT), Judgment Line Orientation (JLO), Rey-Osterrieth Complex Figure (ROCF) (copy and recall at 3 and 30 min), Free and Cued Selective Reminding Test (FCSRT), verbal fluencies (animals and words beginning with “p” in 1 min), Stroop Color Word Interference Test, and Tower of London (ToL). These tests were chosen because they examine the main cognitive domains (i.e., attention and executive functioning, memory, language, visuospatial function) and because solid normative data are available in our setting (23, 24).

Likewise, we evaluated fatigue and depression using the Fatigue Severity Scale (FSS) (25) and the Beck Depression Inventory (BDI) (26). We analyzed the following variables: gender, laterality, years of formal schooling, age, disease progression time in years, clinical form of MS, and disability, as measured on the Expanded Disability Status Scale (EDSS) (27). Patients also completed the 3-min version of the PASAT, but this test was not included in the assessment protocol given the lack of normative data in our setting.

Raw scores on each of the neuropsychological tests were converted to scaled scores (mean 10, SD 3) and adjusted by age, years of schooling, and gender using normative data from the NEURONORMA project. This is a normative study using cognitive tests conducted in Spain and including individuals between 18 and 90 years; it is similar in design to Mayo’s Older American Normative Studies (23), and featured detailed evaluations of 535 healthy subjects. The NEURONORMA project recruited individuals from different regions of Spain, set strict inclusion and exclusion criteria, and made use of a thorough protocol to ensure inclusion of cognitive healthy subjects in a representative sample. Following these authors’ recommendations, a scaled score ≤5 (percentile ≤5) was considered impaired in our study (23).

### Definition of Cognitive Domain and Cognitive Impairment

To define impairment in each cognitive domain, we used normative data and within-subject comparison of tests following the criteria listed in Table 2 (28, 29).

**Table 2:** Criteria for defining impairment in each cognitive domain.

Domain	Criteria (at least one of the following)
Attention and executive function	– TMT: impairment on part B, normal results on part A – ToL: total number of moves and/or number of correct moves – Stroop test: impaired scoring on part C, normal results on parts A and B – Raw backward digit verbal or visual span scores ≥ 3 below verbal or visual forward digit span scores. – Impaired formal fluency but normal semantic fluency – Impaired formal and semantic fluencies with normal BNT scores

IPS	– Impaired SDMT scores – Impaired TMT part A scores

Visuospatial function	– Impaired JLO scores – Impaired ROCF scores (copy accuracy)

Memory	– Impaired FCSRT scores (total recall or total delayed recall) with normal BNT scores – ROCF: impaired scores for 30-minute recall but normal scores for copy

Language	– Impaired BNT scores – Impaired semantic fluency with normal formal fluency

*BNT, Boston Naming Test; FCSRT, Free and Cued Selective Reminding Test; IPS, information processing speed; JLO, Judgment Line Orientation; ROCF, Rey-Osterrieth Complex Figure; SDMT, Symbol Digit Modalities Test; ToL, Tower of London; TMT, Trail Making Test*.

Patients displaying deficits in two or more cognitive domains were considered to have cognitive impairment associated with MS (CI-MS) (30). In contrast, patients with no impairment in any of the cognitive domains were regarded as cognitively healthy (CH-MS).

### Description of the Sample

**Figure 1:**
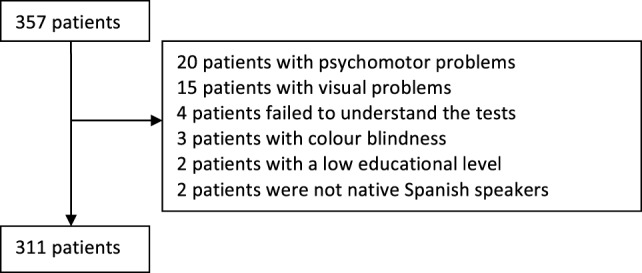
Flowchart of patients included in the study.

Our study included 357 patients with a mean age of 47.35 ± 10.26 years; 245 participants (68.6%) were women. Participants had a mean of 15.05 ± 3.69 years of schooling and their mean score on the EDSS was 3.25 ± 2.16 points. Regarding clinical forms, 251 patients (70.3%) had RRMS, 76 (21.3%) had SPMS, and 30 (8.4%) had PPMS. Only patients who completed the cognitive assessment were considered in our analysis; the rest were excluded. Reasons for not completing the assessment were as follows: fatigue, visual alterations, dysfunction of the dominant hand (tremor, motor impairment, sensory alterations, …); the patient’s deciding to stop for no apparent reason; and not being a native Spanish speaker, which may have yielded poorer results on tests with a verbal component (Figure 1). The final sample consisted of 311 patients, 213 of whom were women (68.5%), with a mean age of 46.39 ± 9.54 years. Table 3 shows the descriptive results of the initial and final samples. Differences in the EDSS scores for the initial and final samples were statistically significant (*P* = 0.043); there were no differences regarding age, gender, years of schooling, or clinical form of MS. Table 4 summarizes the main demographic and clinical data broken down by clinical form.

**Table 3:** Demographic results of the initial (*n* = 357) and final samples (*n* = 311).

	Initial (*n* = 357)	Final (*n* = 311)
Age, years (mean ± SD) [interquartile range]	47.35 ± 10.26 [40–54]	46.39 ± 9.54 [39–53]

Sex, women (%)	245 (68.5)	213 (68.5)

Years of schooling (mean ± SD) [interquartile range]	15.05 ± 3.69 [12–18]	15.45 ± 3.46 [12–18]

EDSS score (mean ± SD) [interquartile range]	3.25 ± 2.16 [1.5–5.0]	2.93 ± 1.95 [1.5–4.0]

Disease duration in years (mean ± SD) [interquartile range]	14.54 ± 6.99 [9.0–20.0]	14.08 ± 6.91 [9.0–19.0]

*EDSS, Expanded Disability Status Scale*.

**Table 4:** Clinical and demographic data of the final sample (*n* = 311).

	RRMS (236)	SPMS (52)	PPMS (23)	Total (311)
Age, years (mean ± SD)	44.33 ± 8.62	51.48 ± 8.54	56 ± 10.78	46.39 ± 9.54

Sex, women (%)	169 (71.6)	33 (63.5)	11 (47.8)	213 (68.5)

Years of schooling (mean ± SD)	15.82 ± 3.18	14.27 ± 3.98	14.35 ± 3.82	15.45 ± 3.46

EDSS score (mean ± SD)	2.16 ± 1.36	5.54 ± 1.38	5.00 ± 1.54	2.93 ± 1.95

Disease duration in years (mean ± SD)	13.07 ± 6.93	19.21 ± 4.94	12.87 ± 5.37	14.08 ± 6.91

*EDSS, Expanded Disability Status Scale; RRMS, relapsing-remitting MS; SPMS, secondary progressive MS; PPMS, primary progressive MS*.

### Statistical Analysis

Statistical analysis was conducted using SPSS Statistics 20.0 by IBM^®^. Results are expressed as either frequencies (percentages) or means ± SD. To compare qualitative and quantitative variables between two groups, we used the chi-square test and the *t* test, as appropriate. Using the “Enter method,” we created a binary logistic regression model to evaluate the factors associated with presence of cognitive impairment. The factors entered in the model were age, progression time, EDSS score, clinical form of MS, and years of schooling. Results were expressed as odds ratios with 95% confidence intervals (95% CI).

Principal component analysis with oblimin rotation was used to identify patterns of clustering of cognitive tests; we extracted the components with an eigenvalue ≥1. We used scaled scores from the cognitive tests included in the study and raw scores from the scales for depression and fatigue. Tests with coefficients ≥0.3 were regarded as factors of each component. We subsequently calculated scores for each of the identified clusters using the Anderson–Rubin test and calculated correlations with progression time, adjusted for age and clinical form of MS. We also conducted an analysis of variance (ANOVA) and Tukey’s *post hoc* test to assess differences between clinical forms with regard to the analyzed components. The Levene test was used to check for homogeneity of variances.

Statistical significance was set at *P* < 0.05.

## Results

### Frequency of CI-MS and Impaired Cognitive Domains

Of the 311 analyzed patients, 129 (41.5%) were categorized in the CI-MS group and 69 (22.2%) in the CH-MS group; 113 patients (36.6%) showed deficits in a single cognitive domain. The most frequently impaired domains were attention (57; 50.4%), information processing speed (30; 26.5%), memory (14; 12.4%), visuospatial function (10; 8.8%), and language (2; 1.8%). In the 129 patients displaying deficits in two cognitive domains, the most frequent combinations were attention and information processing speed (19; 33.3%), attention and memory (11; 19.3%), information processing speed and memory (11; 19.3%), attention and visuospatial function (5; 8.8%), and attention and language (3; 5.3%). At least three cognitive domains were affected in 72 patients (23.2%).

According to the logistic regression analysis, only EDSS scores and years of schooling were independent predictors of CI-MS (Table 5). The correct classification rate for this model was 65.6%.

**Table 5:** Results of the binary logistic regression analysis for predicting presence of CI-multiple sclerosis.

	β_0_	*P*-value	Odds ratio (95% CI)
Age	−0.024	0.128	0.976 (0.946–1.007)
Progression time	0.022	0.288	1.022 (0.982–1.065)
**EDSS score (mean ± SD)**	**0.277**	**0.003**	**1.319 (1.100–1.582)**
**Years of schooling**	−**0.100**	**0.008**	**0.905 (0.840–0.974)**
RRMS	−0.427	0.443	0.652 (0.219–1.942)
SPMS	−0.549	0.325	0.577 (0.193–1.724)
Constant	1.593	0.214	4.918

*CI-MS, cognitive impairment associated to multiple sclerosis; EDSS, Expanded Disability Status Scale; RRMS, relapsing-remitting MS; SPMS, secondary progressive MS*.

*Statistically significant values (p < 0.05) are shown in bold*.

### Principal Component Analysis

The Kaiser–Meyer–Olkin measure of sampling adequacy was 0.870. Results from the Bartlett test were significant (chi-square: 5838; *P* < 0.001). Rotation converged in 10 iterations. We obtained seven components, which explained 64.6% of the variance (Table 6; Table S1 in Supplementary Material). Component 1 included Stroop test part A, TMT part B, Stroop test part B, Stroop test part C, SDMT, backward Corsi block-tapping test, ROCF (time to copy), Corsi block-tapping test, and formal fluency; it accounted for 31.2% of the variance. Component 2 consisted of ToL (total number of moves, number of correct moves, execution time, and resolution time) and explained 8.1% of the variance. Component 3 was formed by different FCSRT scores (free and total recall, total recall, trial 1 free recall, delayed total recall, delayed free recall), BNT, and semantic fluency. Component 4 included time to start ToL test, time to copy the ROCF, and execution and resolution times for the ToL test. Component 5 included the scales for fatigue and depression (FSS and BDI). Component 6 was formed by 3- and 30-min recall of the ROCF, copy of the ROCF, JLO, and visuographic memory of the ROCF. Finally, component 7 included forward Corsi block-tapping test, backward Corsi block-tapping test, JLO, and forward and backward digit span.

**Table 6:** Principal component analysis: rotated component matrix showing loadings for each test and each of the components.

	1	2	3	4	5	6	7
Forward digit span	0.122	0.005	0.052	0.046	−0.103	−0.019	−**0.737**
Backward digit span	0.221	0.052	0.225	0.084	−0.010	0.019	−**0.634**
Forward Corsi test	**0.493**	0.031	−0.089	−0.069	0.057	−0.163	−**0.421**
Backward Corsi test	**0.544**	0.038	−0.010	−0.082	0.058	−0.126	−**0.313**
TMT, part A	**0.777**	0.046	−0.030	−0.020	0.017	−0.128	0.095
TMT, part B	**0.627**	0.173	0.061	0.014	0.059	−0.157	−0.026
SDMT	**0.683**	0.001	0.169	−0.023	0.093	−0.125	0.016
BNT	0.112	0.092	**0.397**	0.170	0.057	−0.068	−0.028
JLO	−0.002	0.089	0.013	0.092	0.210	−**0.442**	−**0.312**
ROCF copy (accuracy)	0.064	0.029	−0.022	0.134	0.035	−**0.631**	0.027
ROCF copy (time)	**0.497**	0.093	−0.082	−**0.342**	−0.004	−0.279	0.189
ROCF 3-minute recall	0.005	0.010	0.113	−0.105	−0.052	−**0.867**	0.049
ROCF 30-minute recall	−0.012	0.037	0.101	−0.048	−0.077	−**0.867**	0.039
ROCF recognition	−0.072	0.085	0.193	0.013	−0.245	−**0.433**	−0.151
FCSRT 1-minute free recall	0.020	−0.015	**0.811**	−0.086	0.032	0.016	−0.185
FCSRT total free recall	0.035	−0.034	**0.890**	−0.066	0.040	−0.005	−0.157
FCSRT total recall	−0.094	0.070	**0.860**	−0.041	−0.040	−0.003	−0.051
FCSRT delayed free recall	0.120	−0.140	**0.516**	−0.011	−0.013	−0.119	0.169
FCSRT delayed total recall	−0.005	0.030	**0.697**	−0.029	−0.040	−0.142	0.130
Semantic fluency	0.195	0.197	**0.309**	0.156	0.002	0.026	0.283
Formal fluency	**0.438**	0.108	0.205	0.197	−0.075	−0.048	0.019
Stroop test part A	**0.842**	−0.086	0.088	0.051	−0.109	0.144	−0.048
Stroop test part B	**0.757**	−0.001	0.093	0.002	−0.164	0.122	−0.065
Stroop test part C	**0.737**	0.066	−0.017	0.061	−0.102	0.084	−0.189
ToL correct moves	−0.049	**0.868**	−0.007	0.249	0.003	−0.027	−0.007
ToL total moves	−0.047	**0.935**	−0.065	0.034	−0.038	−0.067	0.075
ToL initiation time	−0.050	−0.036	0.096	−**0.882**	0.005	0.068	0.049
ToL execution time	0.136	**0.800**	0.028	−**0.303**	−0.002	−0.037	−0.056
ToL problem-solving time	0.118	**0.685**	0.113	−**0.540**	0.031	0.027	−0.108
FSS	−0.019	−0.065	0.056	0.073	**0.845**	−0.020	0.045
BDI	−0.061	0.049	0.013	−0.081	**0.827**	0.092	−0.001
% of variance	31.2	8.12	7.57	5.06	4.80	4.16	3.67

*Coefficients > 0.3 are shown in bold*.

*BDI, Beck Depression Inventory; BNT, Boston Naming Test; FCSRT, Free and Cued Selective Reminding Test; FSS, Fatigue Severity Scale; IPS, information processing speed; JLO, Judgment Line Orientation; ROCF, Rey-Osterrieth Complex Figure; SDMT, Symbol Digit Modalities Test; ToL, Tower of London; TMT, Trail Making Test*.

### Cognitive Impairment by Clinical Form of MS and Disease Progression Time

Frequency of CI-MS varied among the clinical forms of MS: cognitive impairment was observed in 36% of the patients with RRMS, 57.7% of those with SPMS, and 60.9% of those with PPMS (*P* = 0.002). ANOVA found statistically significant differences in components 2 (higher-order executive functioning) (*F* = 4.301; *P* = 0.014) and 3 (verbal episodic memory and language) (*F* = 4.650; *P* = 0.010). The *post hoc* test revealed significant differences between RRMS and PPMS with regard to component 2 (*P* = 0.042) and between RRMS and SPMS with regard to component 3 (*P* = 0.008); mean of the components was higher for RRMS in both cases. We found no significant differences between clinical forms of MS in the rest of components (*P* > 0.05) (Table S2 in Supplementary Material). Likewise, we calculated partial correlations between the estimated components and disease progression time, adjusted for age, years of schooling, and clinical form, and observed a weak correlation between disease progression time and components 3 (*r* = –0.161, *P* = 0.005) and 6 (*r* = 0.132, *P* = 0.021). Correlations with the remaining components were not significant (*P* > 0.05).

## Discussion

Our study showed a frequency of cognitive impairment of 41.5%, which is in line with frequencies reported by most studies (1–3, 31). To minimize the risk of false positives, we used a strict definition of cognitive impairment: an age-, gender-, and education-adjusted scaled score ≤5 (equivalent to −1.67 SDs) in at least two cognitive domains. This criterion was also chosen to be consistent with recommended criteria for the normative data of the tests used in our study (23) and to avoid the possibility of false positives because the use of a large battery with multiple tests. If we had used a less strict criterion (for example, defining cognitive impairment as deficits in one cognitive domain only), 77.8% of the sample would have been classified as cognitively impaired. As demonstrated by Fisher et al. (31), there is a large heterogeneity in the definition of cognitive impairment across different studies published in the literature. Our study is not performed using a population-based design, so frequency of cognitive impact should be interpreted in its context. However, our definition of a group of patients with cognitive impairment allow us to draw conclusions about cognitive domains most frequently impaired as well as potential associated factors. In this regard, according to our analysis, attention was the most frequently affected domain, whether alone or in combination with other impairments, especially those affecting memory and information processing speed.

The two factors independently associated with cognitive impairment were higher EDSS scores and a lower educational level. Age and disease progression time, in contrast, showed no significant association with cognitive impairment in logistic regression models. This means that cognitive impairment may be partially explained by the degree of disability, but it is not associated with age or disease progression time. This finding suggests that there may be a subgroup of patients with greater clinical severity. Likewise, the protective effects of a higher educational level support the cognitive reserve hypothesis; accordingly, recent studies suggest that education is a protective factor against cognitive impairment (13, 32).

One of the main findings from our study comprised the six cognitive clusters identified by the principal component analysis. The first component consists of tests assessing attention and basic executive function, more specifically divided attention, information processing speed, focused attention, vigilance, and visuospatial working memory. The second component includes the ToL test, which is linked to high-order planning and executive function. Component 3 is formed by verbal memory and language. However, taking into account the importance of verbal memory in MS and the influence of verbal memory in language tasks (verbal fluency, for instance), this component 3 may be probably more associated to verbal memory than language (33). Component 4 includes tests evaluating time to complete executive and visuospatial tasks. Component 6 includes visuospatial function, and component 7 consists of tests of basic attention and verbal and visual working memory. Although the first cluster explains the greatest percentage of the variance, our results suggest that multiple components are present in addition to impairments in attention and information processing speed. In light of the above, information processing speed, understood as the speed at which a person can process information and evaluated based on the amount of time a person takes to complete a cognitive task, would only be contemplated by components 1 and 4. The SDMT, which some studies have found to be sensitive to impairments in information processing speed (34), may be useful for evaluating the first component (attention and basic executive function) but not as a measure of heterogeneity of cognitive profiles in MS given that information processing speed is barely correlated with the other components. Our results suggest that slow information processing speed is a relevant factor in some cognitive deficits associated with MS, although it does not appear to be the key element explaining the rest of the associated cognitive deficits.

To our knowledge, principal component analysis in cognitive assessment in MS has only been performed in two series of patients using MACFIMS battery, including SDMT, COWAT, PASAT, JLO, California Verbal Learning Test-II, DKFEFS-Sorting test and Description Score and Brief Visuospatial Memory Test (BVMT) (20, 21). In the most recent work by Migliore et al., four components were found including visuospatial memory and processing speed (BVMT and SDMT), working memory and visuospatial function (PASAT and JLO), executive functioning (D-KEFS), and verbal memory (CVLT). Thus, we observed some more components, probably because our study included some more cognitive tests and functions, and a larger sample. Intriguingly, in our study SDMT did not form a cluster with visual memory, two dissociate components of attention/executive function were identified (planning in component 2 and attention, basic executive functioning and working memory in components 1 and 7), and visuospatial function constituted a separate component. Although our study found seven components, an important part of the variance was explained by components that are usually assesses in MS, which confirms the usefulness of current comprehensive scales such as MACFIMS and others for the cognitive evaluation in MS. However, future studies would be interesting to validate the protocol included in our study (mainly, tests provided by NEURONORMA battery) in comparison to current gold standard scales in MS.

Another interesting finding is the presence of a component limited to fatigue and depression. We decided to include fatigue and depression in the principal component analysis in order to exclude their potential influence in cognitive testing. The correlation observed between fatigue and depression has been previously shown and may be indicative of common pathophysiological mechanisms (35). Importantly, neither depression nor fatigue had a significant impact on any of the remaining clusters. The association between fatigue and depression, on the one hand, and cognitive function, on the other, is controversial; some studies have found an association between fatigue and memory, information processing speed, working memory, language, and especially attention and vigilance (36, 37). Our results show a potential, but weak correlation between scales assessing fatigue and depression and some cognitive tests; but fatigue and depression have no significant impact on any of the affected cognitive domains in MS. The existence of this cluster supports the hypothesis that fatigue and depression are associated: although the causes of MS-associated fatigue are not completely clear, there are several proposed mechanisms, including muscle problems, proinflammatory cytokine production, conduction disorders, focal lesions, and psychological factors associated with depression (38). However, the association between fatigue and depression may in fact indicate inability by the scales used in this study to define and distinguish between these two entities. In this regard, the scale used in our study for depression might led to an overdiagnosis of depression, and other scales have also been suggested (39). Further studies should be designed specifically to evaluate these aspects.

Regarding the comparison between clinical forms of MS, we found cognitive impairment to be more frequent in patients with progressive forms, both SPMS and PPMS; our results are in line with recent literature (13, 40, 41). However, other studies found lower frequencies of cognitive impairment in patients with PPMS (14, 15). Interestingly, comparisons between cognitive components show similar cognitive profiles for all three clinical forms of MS, although significant differences are also present. We observed more severe impairments in planning and complex executive function in PPMS than in RRMS, and more severe deficits in verbal memory and language in SPMS than in RRMS. Likewise, only two of the components (those related to memory, language, and visuospatial function) were correlated, weakly in both cases, with disease progression time. Differences between the RRMS and SPMS groups in the memory and language component, and the correlation between this component and progression time, may suggest that these functions, and not those associated with other cognitive domains, deteriorate the most throughout disease progression. However, longitudinal studies are necessary to confirm this hypothesis. Furthermore, neuroimaging studies correlating MRI findings with cognitive clusters found in our study would be of interest. In this regard, recent studies have associated cognitive impairment with cortical lesions using high field MRI (42–45). Interestingly, executive dysfunction has been associated with the presence of intracortical or subpial lesions and a diffuse degeneration from outer cortical lesions, while other cognitive functions to degeneration of deeper cortical layers. Because some differences in the pattern of degeneration of cortical layers have been observed between relapsing and progressive stages of the disease, these MRI findings could help to explain differences in cognitive patterns between MS forms (45).

Our study has some limitations. The length of cognitive assessment may have resulted in a selection bias: some of the patients (those with higher EDSS scores) were not included in our analysis, and our study was not population based. The frequency of cognitive impairment in our sample may, therefore, be higher than observed. However, given the characteristics of our study, we decided to select only those patients whose cognitive assessment results were not affected by such other factors as motor disorders or coordination problems. Furthermore, given the cross-sectional design of our study, any results having to do with the potential progression of cognitive impairment should be interpreted with caution and reassessed in longitudinal studies.

In conclusion, the frequency of cognitive impairment in our sample was 41.5%. Although cognitive impairment was more frequent in patients with progressive forms of MS, the only factors independently associated with presence of cognitive impairment were the degree of disability and the educational level; no independent associations were found between cognitive impairment and age, progression time, or clinical form of MS. Our results suggest that cognitive performance in MS is described by multiple cognitive domains and profiles. A first component including attention and basic executive functioning entailed the greater percentage of variance (32%), and other components represented a 4–8% each one. These findings reflect a significant variability of cognitive deficits in MS, a pattern that is consistent with the heterogeneity of clinical manifestations of the disease.

## Ethics Statement

*Research Involving Human Participants*: All procedures performed were in accordance with the ethical standards of the institutional research committee and with the 1964 Helsinki declaration and its later amendments. *Informed Consent*: Written informed consent was obtained from all individual participants included in the study.

## Author Contributions

JAM-G: study concept and design; literature search; interpretation of data; statistical analysis of data; and writing of the manuscript. AC-M: acquisition of data; literature search; interpretation of data; statistical analysis of data; and writing of the manuscript. MV-S: acquisition of data; literature search. CO-G: acquisition of data; interpretation of data; and study supervision. VP: acquisition of data; literature search; and statistical analysis. PM: acquisition of data; study concept and design; and interpretation of data. TM-R: study supervision; interpretation of data; critical revision of the manuscript for important intellectual content. JM-G: study concept and design; study supervision; interpretation of data; and critical revision of the manuscript for important intellectual content.

## Conflict of Interest Statement

The authors declare that the research was conducted in the absence of any commercial or financial relationships that could be construed as a potential conflict of interest. The reviewer RL and handling Editor declared their shared affiliation.
