# Supplementary material for: Functional Components of Cognitive Impairment in Multiple Sclerosis: A Cross-Sectional Investigation
Source: Front Neurol. 2017 Nov 28;8:643. doi: 10.3389/fneur.2017.00643 (PMC5712315; doi:10.3389/fneur.2017.00643)
Supplement: Supplementary file 1 [file Table_1.DOCX]

**SUPPLEMENTARY INFORMATION**

| **Table S1.** Analysis of variance. Mean variance of each component for each clinical form of MS | | | | |
| --- | --- | --- | --- | --- |
|  | RRMS | SPMS | PPMS | F (*P*-value) |
| Component 1 | 0.04 ± 0.95 | –0.24 ± 1.18 | 0.06 ± 1.00 | 1.930 (0.147) |
| Component 2 | 0.08 – 0.94^b^ | –0.20 ± 1.16 | –0.43 ± 1.03^b^ | 4.301 (0.014) |
| Component 3 | 0.08 ± 0.97^a^ | –0.36 ± 1.08^a^ | –0.095 ± 0.83 | 4.650 (0.010) |
| Component 4 | –0.05 ± 0.98 | 0.03 ± 0.96 | 0.45 ± 1.12 | 2.733 (0.067) |
| Component 5 | –0.01 ± 1.05 | 0.11 ± 0.81 | –0.12 ± 0.70 | 0.520 (0.595) |
| Component 6 | –0.05 ± 1.01 | 0.20 ± 0.96 | 0.14 ± 0.92 | 1.767 (0.173) |
| Component 7 | 0.06 ± 1.01 | –0.10 ± 1.04 | –0.38 ± 0.64 | 2.483 (0.085) |
| ^a^ RRMS vs SPMS, *P* < 0.05.  ^b^ RRMS vs PPMS, *P* < 0.05. | | | | |

| **Table S2.** Correlation matrix showing correlations between components of the principal component analysis | | | | | | | |
| --- | --- | --- | --- | --- | --- | --- | --- |
| Components | 1 | 2 | 3 | 4 | 5 | 6 | 7 |
| 1 | 1 | 0.344 | 0.413 | –0.035 | –0.154 | –0.323 | –0.243 |
| 2 |  | 1 | 0.226 | –0.025 | –0.010 | –0.370 | –0.089 |
| 3 |  |  | 1 | 0.061 | –0.116 | –0.370 | –0.062 |
| 4 |  |  |  | 1 | 0.013 | 0.010 | –0.019 |
| 5 |  |  |  |  | 1 | –0.007 | 0.010 |
| 6 |  |  |  |  |  | 1 | 0.117 |
| 7 |  |  |  |  |  |  | 1 |
